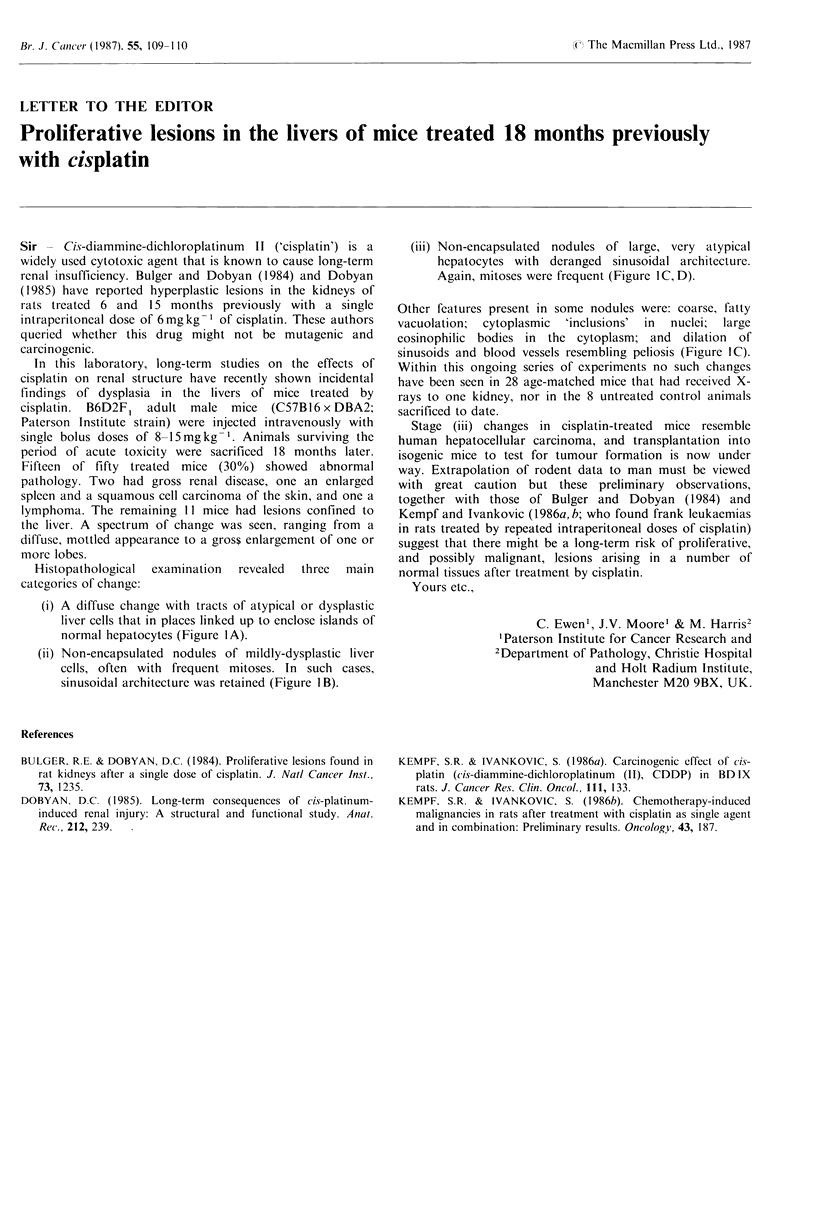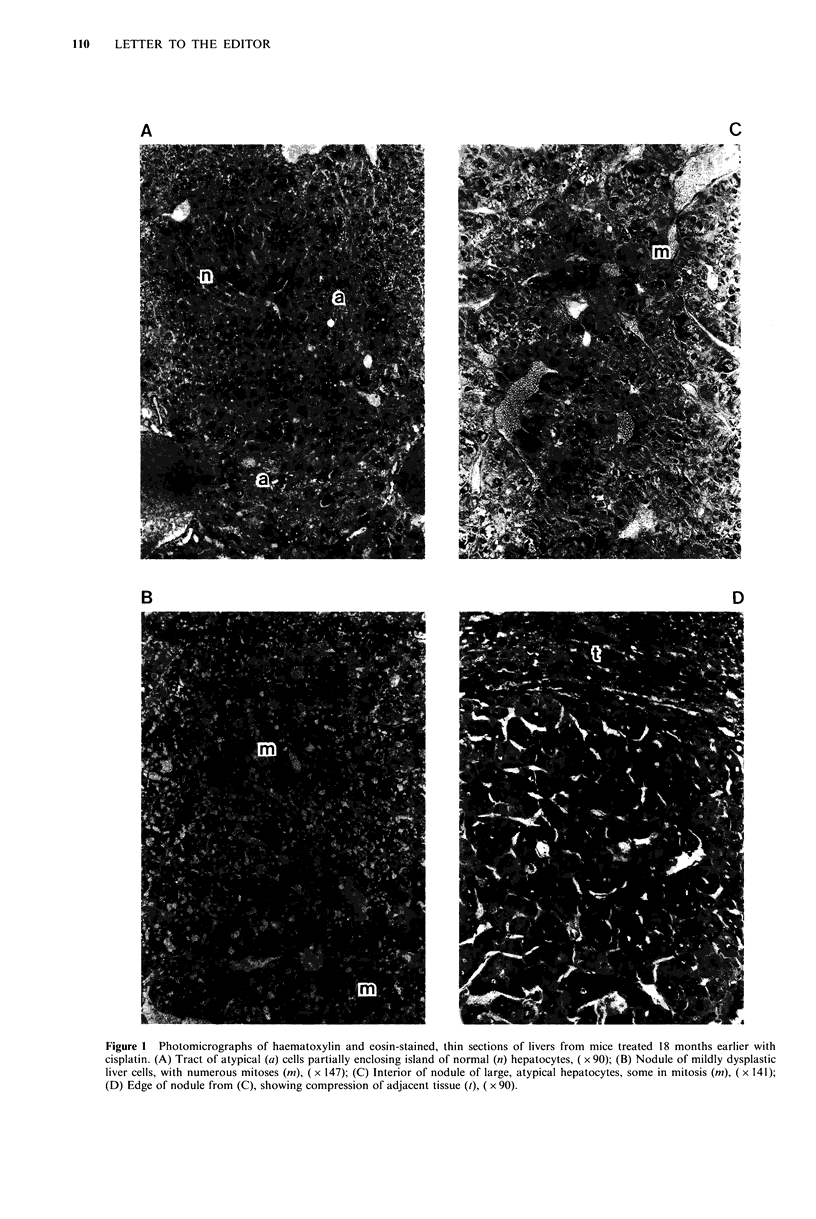# Proliferative lesions in the livers of mice treated 18 months previously with cisplatin.

**DOI:** 10.1038/bjc.1987.23

**Published:** 1987-01

**Authors:** C. Ewen, J. V. Moore, M. Harris

## Abstract

**Images:**


© The Macmillan Press Ltd., 1987

### LETTER TO THE EDITOR

### Proliferative lesions in the livers of mice treated 18 months previously with *cis*platin

**Sir** – *Cis*-diammine-dichloroplatinum II ('cisplatin') is a widely used cytotoxic agent that is known to cause long-term renal insufficiency. Bulger and Dobyan (1984) and Dobyan (1985) have reported hyperplastic lesions in the kidneys of rats treated 6 and 15 months previously with a single intraperitoneal dose of $6\,mg\,kg^{-1}$ of cisplatin. These authors queried whether this drug might not be mutagenic and carcinogenic.

In this laboratory, long-term studies on the effects of cisplatin on renal structure have recently shown incidental findings of dysplasia in the livers of mice treated by cisplatin. $B6D2F_1$ adult male mice (C57B16 x DBA2; Paterson Institute strain) were injected intravenously with single bolus doses of $8–15\,mg\,kg^{-1}$. Animals surviving the period of acute toxicity were sacrificed 18 months later. Fifteen of fifty treated mice (30%) showed abnormal pathology. Two had gross renal disease, one an enlarged spleen and a squamous cell carcinoma of the skin, and one a lymphoma. The remaining 11 mice had lesions confined to the liver. A spectrum of change was seen, ranging from a diffuse, mottled appearance to a gross enlargement of one or more lobes.

Histopathological examination revealed three main categories of change:

(i) A diffuse change with tracts of atypical or dysplastic liver cells that in places linked up to enclose islands of normal hepatocytes (Figure 1A).

(ii) Non-encapsulated nodules of mildly-dysplastic liver cells, often with frequent mitoses. In such cases, sinusoidal architecture was retained (Figure 1B).

(iii) Non-encapsulated nodules of large, very atypical hepatocytes with deranged sinusoidal architecture. Again, mitoses were frequent (Figure 1C, D).

Other features present in some nodules were: coarse, fatty vacuolation; cytoplasmic 'inclusions' in nuclei; large eosinophilic bodies in the cytoplasm; and dilation of sinusoids and blood vessels resembling peliosis (Figure 1C). Within this ongoing series of experiments no such changes have been seen in 28 age-matched mice that had received X-rays to one kidney, nor in the 8 untreated control animals sacrificed to date.

Stage (iii) changes in cisplatin-treated mice resemble human hepatocellular carcinoma, and transplantation into isogenic mice to test for tumour formation is now under way. Extrapolation of rodent data to man must be viewed with great caution but these preliminary observations, together with those of Bulger and Dobyan (1984) and Kempf and Ivankovic (1986*a*, *b*; who found frank leukaemias in rats treated by repeated intraperitoneal doses of cisplatin) suggest that there might be a long-term risk of proliferative, and possibly malignant, lesions arising in a number of normal tissues after treatment by cisplatin.

Yours etc.,

C. Ewen[1], J.V. Moore[1] & M. Harris[2]
[1]Paterson Institute for Cancer Research and
[2]Department of Pathology, Christie Hospital
and Holt Radium Institute,
Manchester M20 9BX, UK.

### References

BULGER, R.E. & DOBYAN, D.C. (1984). Proliferative lesions found in rat kidneys after a single dose of cisplatin. *J. Natl Cancer Inst.*, **73**, 1235.

DOBYAN, D.C. (1985). Long-term consequences of *cis*-platinum-induced renal injury: A structural and functional study. *Anat. Rec.*, **212**, 239.

KEMPF, S.R. & IVANKOVIC, S. (1986*a*). Carcinogenic effect of *cis*-platin (*cis*-diammine-dichloroplatinum (II), CDDP) in BD IX rats. *J. Cancer Res. Clin. Oncol.*, **111**, 133.

KEMPF, S.R. & IVANKOVIC, S. (1986*b*). Chemotherapy-induced malignancies in rats after treatment with cisplatin as single agent and in combination: Preliminary results. *Oncology*, **43**, 187.

**Figure 1** Photomicrographs of haematoxylin and eosin-stained, thin sections of livers from mice treated 18 months earlier with cisplatin. (A) Tract of atypical (*a*) cells partially enclosing island of normal (*n*) hepatocytes, ( × 90); (B) Nodule of mildly dysplastic liver cells, with numerous mitoses (*m*), ( × 147); (C) Interior of nodule of large, atypical hepatocytes, some in mitosis (*m*), ( × 141); (D) Edge of nodule from (C), showing compression of adjacent tissue (*t*), ( × 90).